# Familial Normokalemic Periodic Paralysis Associated With Mutation in the *SCN4A* p.M1592V

**DOI:** 10.3389/fneur.2018.00430

**Published:** 2018-06-07

**Authors:** Chao Fu, Zhenyu Wang, Libo Wang, Jia Li, Qiuling Sang, Jiajun Chen, Ling Qi, Hui Jin, Xiaoyang Liu

**Affiliations:** ^1^Department of Neurosurgery, China-Japan Union Hospital of Jilin University, Changchun, China; ^2^Department of Neurology, China-Japan Union Hospital of Jilin University, Changchun, China; ^3^Department of Pathophysiology, Jilin Medical University, Jilin, China

**Keywords:** normokalemic periodic paralysis, *SCN4A*, mutations, pedigree, respiratory muscle paralysis

## Abstract

Periodic paralysis (PP) is an uncommon inherited disorder causing recurrent episodes of muscle weakness, with an incidence of 0.001%. Normokalemic periodic paralysis (NormoKPP) as the rarest subtype of PP contains both familial and sporadic. Familial NormoKPP caused by the p.M1592V mutation of the skeletal muscle sodium channel alpha subunit (*SCN4A*) gene is rarely reported. Only three pedigrees of NormoKPP related to mutations in the *SCN4A* p.M1592V have been previously reported. We herein presented a family case of NormoKPP associated with the *SCN4A* p.M1592V mutation, in which respiratory muscle paralysis occurred in the proband while not in his children. Moreover, we conducted a thorough literature review. To our knowledge, this is the first report of respiratory muscle paralysis as a symptom of NormoKPP associated with mutation in the *SCN4A* p.M1592V.

## Introduction

Periodic paralysis (PP) is a rare muscle disease characterized by recurrent muscle weakness, occurring at a rate of 0.001% (1). Generally, based on serum potassium levels, PP was divided into three subtypes, including hypokalemia, normokalemia, and hyperkalemia. Normokalemic periodic paralysis (NormoKPP) is the rarest subtype of PP, with both familial and sporadic forms. Previously, seven mutations of the skeletal muscle sodium channel alpha subunit (*SCN4A*) gene associated with NormoKPP have been reported. Notably, mutations were enriched at exons 12, 13, and 24 of the *SCN4A* gene, leading to amino acid substitutions including T704M, M1592V, V781I, R675G, R675W, R675Q, and R1129Q (2–5). To the best of our knowledge, NormoKPP associated with the p.M1592V mutation has been reported in only three pedigrees (3, 6, 7). In this study, we report a familial NormoKPP associated with the *SCN4A* p.M1592V mutation, and reviewed the literature.

## Case presentation

The proband, a 45-year-old man, was admitted to hospital with increasing frequency and severity of recurrent muscular paralysis. Past history includes diabetes, coronary heart disease, and myocardial infarction. He is currently taking 250 mg of metformin trice a day and 100 mg of aspirin once a day. Periodic weakness lasted for 13 days. First limb flaccid paralysis occurred at the age of 4, lasting for 5–13 days. During this episode, his muscular strength could barely lift his limbs from the bed (3/5), while his proximal limbs were even weaker (2/5). Symptoms are most severe in the mornings. Cold and humid weather were likely to induce an attack. The patient took potassium chloride orally but only to make symptoms worse. Notably, the patient presented with recurrent respiratory muscle paralysis diagnosed on the basis of the following remarks: (a) he became short of breath; (b) intraarterial PaCO2 and PaO2 both decreased slightly; (c) the symptom was relieved with inhaled budesonide; (4) other comorbidity condition related to the symptom was excluded.

Routine laboratory tests found elevated fasting glucose (17.98 mmol/L, normal reference range <6.10 mmol/L), elevated glycosylated hemoglobin (11.40%, normal reference range <6.00%) and creatine kinase (CK) (843.84 U/L, Normal reference range <170 U/L). Blood potassium level fluctuated between 4.3 and 5.1 mmol/L (3.5 and 5.5 mmol/L) during hospitalization.

**Figure 1:**
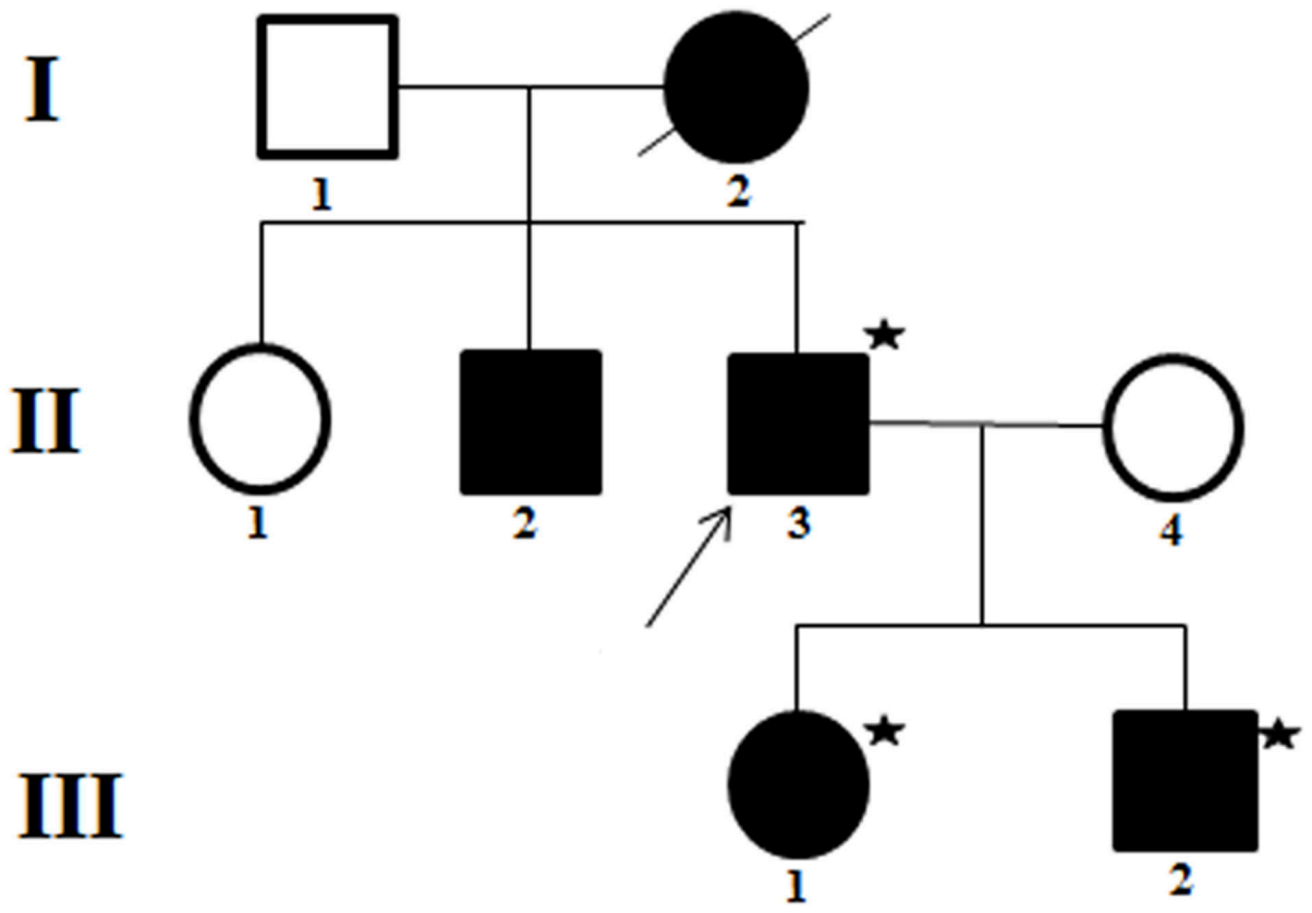
Pedigree of a family presenting with normokalemic periodic paralysis (NormoKPP). The filled symbols indicate individuals with NormoKPP, whereas the open symbols show individual without NormoKPP. The individuals with asterisks were examined by gene sequencing. The arrow indicates the index case.

Multiple family members were involved (Figure 1, Table 1). It is note of that respiratory muscle paralysis occurred in the proband while is not observed in the other members of the family. Large dosage of normal saline could relief the symptoms during the attack, and they was administrated with 250 mg of acetazolamide twice a day during intermittent period.

**Table 1:** Clinical characteristics of normokalemic periodic paralysis related to *SCN4A* p.M1592V mutation.

**Individual annotation**	**II-3**	**III-1**	**III-2**
Age (years) and sex	45, M	23, F	19, M
PP onset (years)	4	3	5
Episode duration (days)	5–13	2–5	2–7
**CLINICAL FEATURES**			
RMP	+	–	–
Muscle strength	2/5	3/5	3/5
Provocative factors	Coldness Humid weather Oral potassium chloride	Coldness Infection Eat bananas	Coldness Exercise Eat bananas
Attack frequency (per year)	2–24	3–15	3–12

*M, male; F, female; RMP, respiratory muscles paralysis*.

**Figure 2:**
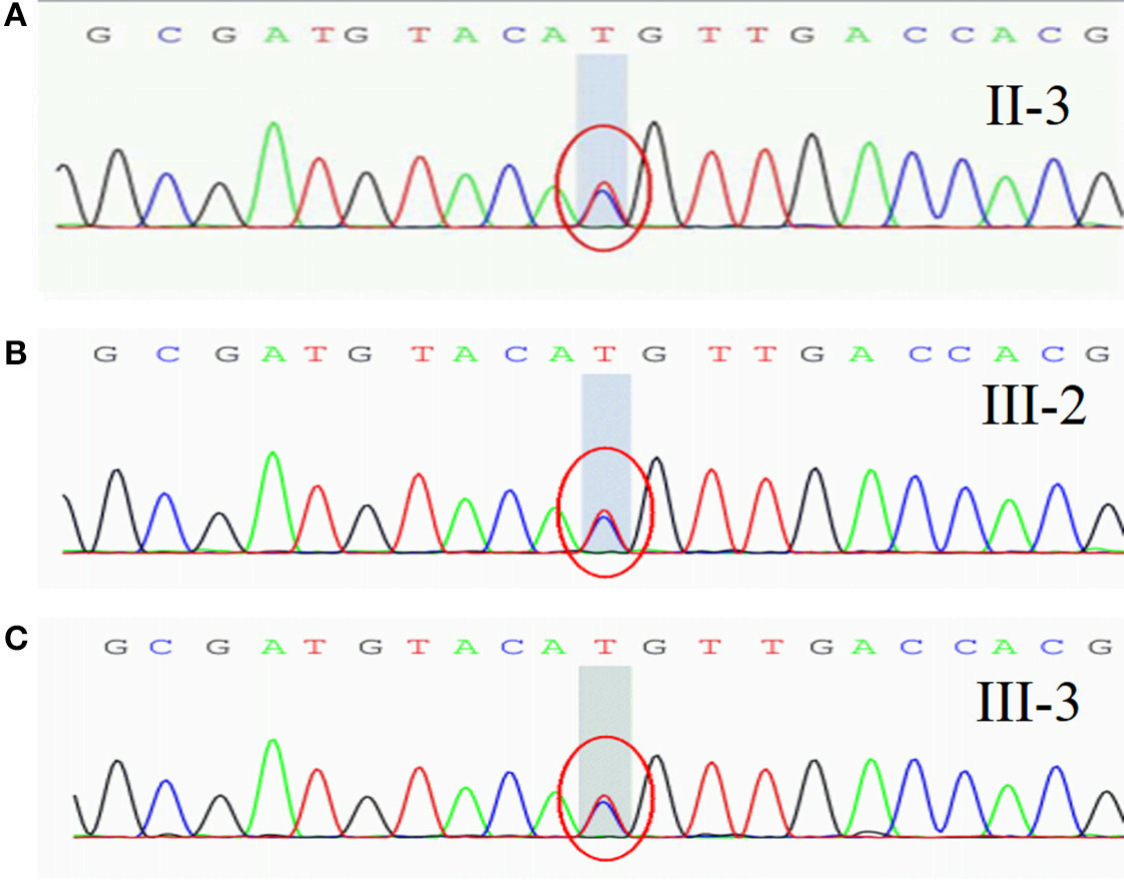
A heterozygous mutation of c.4774A > G in the nucleotide sequence of *SCN4A*, resulting in a change from methionine to valine (p.M1592V).

They refused to do the neurophysiological test. Gene sequencing showed a heterozygous mutation of c.4774A > G in the nucleotide sequence of *SCN4A*. The mutation resulted in a change from methionine to valine (p.M1592V) (Figure 2A). His children had the same heterozygous mutation point in the exon region of the *SCN4A* gene (Figures 2B,C). His other relatives did not accept gene sequencing.

## Discussion

NormoKPP is the rarest subtype of PP, compared with hypokalemic and hyperkalemic PP. It is similar to hyperkalemic PP in the clinical and laboratory features; thus, some authors suggest that NormoKPP may be a variant of HyperKPP rather than a distinct subtype (2, 8). The pathogenesis of NormoKPP has not been fully understood, but the abnormal expression and chronic inactivation of sodium channel genes can be involved (9, 10). Mutations in the *SCN4A* and the skeletal muscle calcium channel gene have been reported to be linked with NormoKPP (11).

Familial NormoKPP was first described in 1961 by Poskanzer and Kerr (12). Such condition with the *SCN4A* p.M1592V mutation is rare. To date, only three families of NormoKPP induced by the p.M1592V mutation have been reported in the literature (Table 2). There were 18 males and 21 females with the age of onset between 1 and 20 years old. Cold and wet weather, season alteration, strenuous exercise, stress, and infection provoked the episode. The episode lasted from 0.5 day to 4 weeks, and no difference between genders existed. Clinical manifestations varied, including mild proximal muscle weakness, calf hypertrophy, percussion myotonia of the thenar muscles, and mild eye lid myotonia. Most patients had a satisfactory prognosis.

**Table 2:** Summary of familial NormoKPP caused by the M1592V mutation of the *SCN4A*.

**Year**	**Authors**	**Sex (M/F)**	**Age (Y)**	**Onset age (Y)**	**Inducement**	**Duration of attack**	**Clinical characteristics during attack**	**Treatment**	**Prognosis**
2008	Xiuhai (3)	8/10	5–54	1–15	Coldness, season alteration, violent exercise, tension	1–17 days	Varying extent of weakness	ACE, saline	Good
2013	Lee (6)	4/6	?	?	Emotional stress, exercise, infection.	1 day−4 weeks	Calf hypertrophy, mild proximal muscle weakness, percussion myotonia of the thenar muscles.	?	?
2014	Shiga (7)	4/4	15–87	2–20	Coldness, exercise, eat see weeds, season alteration	0.5–14 days	Weakness in the arms, the proximal legs and mild eye lid myotonia.	ACE, saline	Good
2018	Present study	2/1	19–45	3–5	Coldness, infection, exercise, humid weather, oral high-potassium foods	2–13 days	Respiratory muscles paralysis, the symptoms became severer in morning, the proximal limbs weakness heavier, attack frequency increases with age.	ACE, saline	Good

*ACE, acetazolamide; F, female; M, male; NA, not available; Y, years*.

In this study, NormoKPP occurred in early childhood or childhood. Consistent with previous findings, severity and frequency of their symptoms increased with age. Notably, the proband had the most severe symptoms in the mornings; such phenomenon might be related to the high blood glucose level, as the blood glucose level fluctuated within 20 mmol/L in the mornings and 10 mmol/L at other times. Notably, respiratory muscle paralysis occurred in the proband while not in his offspring. To our knowledge, this is the first report of such symptom of NormoKPP with *SCN4A* p.M1592V mutation.

## Conclusion

Familial NormoKPP caused by the *SCN4A* p.M1592V mutation is rare, and its clinical features vary. It is the first report of respiratory muscle paralysis as a symptom of such clinical condition which should be kept in mind.

## Ethics statement

This study was carried out in accordance with the recommendations of the Ethics Committee of the China-Japan Union Hospital of Jilin University with written informed consent from all subjects. All subjects gave written informed consent in accordance with the Declaration of Helsinki.

## Author contributions

CF and ZW were responsible for study concept or design, acquisition of data, drafting/revising the manuscript. LW, JL, QS, JC, and LQ were responsible for revising the manuscript. HJ and XL were responsible for study concept or design, drafting/revising the manuscript, and final approval.

### Conflict of interest statement

The authors declare that the research was conducted in the absence of any commercial or financial relationships that could be construed as a potential conflict of interest.
